# Equity evaluation of intensive care unit admission based on comorbidity in hospitalized patients with COVID-19: a cross-sectional analysis

**DOI:** 10.3389/fpubh.2024.1430462

**Published:** 2024-10-28

**Authors:** Yang-Jie Zhu, Jia-Yue Wang, Chen-Nan Wu, Bo-Yang Yu, Tong-Tong Liu, Yuan Liu, Lu-Lu Zhang

**Affiliations:** ^1^Department of Military Health Management, College of Health Service, Naval Medical University, Shanghai, China; ^2^Department of Medical Health Service, General Hospital of Northern Theater Command of PLA, Shenyang, China; ^3^Department of Medical Health Service, 969th Hospital of PLA Joint Logistics Support Forces, Hohhot, China

**Keywords:** COVID-19, intensive care unit, vertical equity, Charlson Comorbidity Index, concentration index

## Abstract

**Background:**

Intensive care unit (ICU) beds played a crucial role in reducing mortality rates of patients with severe COVID-19. The surge in the number of patients led to a shortage of ICU beds, which may have exacerbated inequity of healthcare utilization. However, most attention has been focused on the horizontal equity in healthcare utilization, where individuals with the same needs receive the same services. Vertical equity, where individuals with higher needs receive more healthcare is often neglected, which might overestimate the equity. This study analyzes the vertical equity of ICU utilization among hospitalized patients with COVID-19.

**Methods:**

In total, 18,547 hospitalized patients with COVID-19 in Maryland in 2020 were enrolled in this cross-sectional study. Logistic regression analysis was conducted to determine the independent factors affecting ICU utilization, and the Shapley value decomposition approach was implemented to assess the contribution of the independent variables to disparities in ICU admission. A concentration curve and concentration index were used to assess the vertical equity in healthcare utilization.

**Results:**

ICU utilization by patients with COVID-19 was significantly affected by Charlson Comorbidity Index (CCI), with odds ratios (OR) 1.09 [95% confidence intervals (CI): 1.07–1.10; *p* < 0.001] in univariable analysis and 1.11 (95% CI: 1.09–1.13; *p* < 0.001) in multivariable regression analysis. The most important contributors were household income (32.27%) and the CCI (22.89%) in the Shapley value decomposition analysis. The concentration curve was below the line of equity, and the concentration index was 0.094 (95% CI: 0.076–0.111; *p* < 0.001), indicating that ICU utilization was concentrated among patients with a high CCI. These results were robust for all subgroup analyses.

**Conclusion:**

Among 18,547 hospitalized patients with COVID-19 in Maryland in 2020, ICU utilization was significantly affected by comorbid conditions. The concentration curve and concentration index also indicated that ICU utilization was more concentrated in patients with a higher CCI. The results was consistent with the principle of vertical equity, whereby healthcare resources are more concentrated on COVID-19 patients with higher health needs.

## 1 Introduction

Coronavirus disease 2019 (COVID-19) pandemic has caused approximately 776 million confirmed cases and more than 7.1 million deaths worldwide since late 2019 (1), posing enormous burden on the health system and increasing the inequities experienced by vulnerable populations (2–4). Most patients with COVID-19 have mild symptoms and require only isolation and symptomatic treatment. However, during the initial period of the pandemic, the mortality rate of critically ill patients is extremely high, and almost all deaths occur in these patients (5). A study conducted in the early stage of the COVID-19 involving 44,672 patients with confirmed COVID-19 showed that 81% of infections were mild, whereas 19% progressed to severe or critical illness requiring hospitalization for advanced supportive treatment, and all deaths occurred in the critically ill, who had a mortality rate of up to 49% (6). Other studies have shown that 17–35% of infections require treatment in the intensive care unit (ICU), and 9–19% of patients require mechanical ventilation, with treatment periods ranging from 2 to 4 weeks (7–9).

ICU beds, one of the most important health resources for the treatment of patients with severe COVID-19, play a crucial role in reducing mortality rates. In the initial stages of the pandemic, even countries with world-leading ICU bed capacities, such as the United States, faced acute shortages, with high occupancy rates persisting (10, 11). An Australia national study showed that ICU bed capacity needed to nearly triple to meet patient demand (12). However, ICU beds require a high demand for personnel, space, and equipment. Non-ICU staff need to undergo relevant training or supervision to work in the ICU (13–15). The wards need to be reconfigured from other types of rooms to be operational (16), and equipment needs to be purchased or requisitioned (12), making the expansion of ICU beds extremely difficult (17). During this period, allocating ICU beds based on patient needs to minimize mortality rates was of paramount importance (18). Therefore, the use of ICU beds by hospitalized patients can be used as an indicator of equity in healthcare utilization during a pandemic (19, 20).

Equity in healthcare utilization can be classified into horizontal and vertical forms (21). Horizontal equity means that people with equal needs receive equal treatment irrespective of their sociodemographic characteristics. In contrast, vertical equity is defined as individuals with a higher need receiving more healthcare (22). In previous studies, comorbidities, which refer to pre-existing diseases not directly related to the present hospital admission or outpatient visits, can reflect patients’ health needs through effects on disease burden (21, 23–25). For patients with COVID-19, the Charlson Comorbidity Index (CCI), which assigns different weights to individual comorbidities, is also used to reflect a patient’ health need (26–28).

The United States was the country most severely affected by the COVID-19 pandemic in 2020, with over 19 million confirmed cases and 330,000 deaths (29). The surge in the number of patients led to a shortage of ICU beds, which may have exacerbated inequity of healthcare utilization (30). A multinational consensus believes that addressing the inequity of healthcare utilization is one of the most important measures for ending the COVID-19 public health threat (31). However, there is relatively little research on the impact of health needs on healthcare utilization in patients with COVID-19, namely few studies investigating vertical equity (32). Most attention of researchers and policy-makers has been given to horizontal equity, which might overestimate the equity of healthcare utilization (33). Therefore, studying the vertical equity of ICU utilization among hospitalized patients with COVID-19 in the United States will provide a reference for future responses to similar public health emergencies.

## 2 Materials and methods

### 2.1 Study design and participants

This retrospective study included patients hospitalized with COVID-19 from the State Inpatient Database of Maryland in 2020. In the initial stages of the pandemic, medical resources became scarce, particularly ICU beds. Among the SID database, Maryland It is one of the few states with ICU bed data available. The requirement for informed consent was waived because no personal identification information was collected. This study was approved by the Ethics Committee of the Naval Medical University (No. 2021LL024). The design, implementation, and reporting stages of the study were conducted in accordance with the recommendations of Strengthening the Reporting of Observational Studies in Epidemiology (STROBE) guidelines.

**Figure 1:**
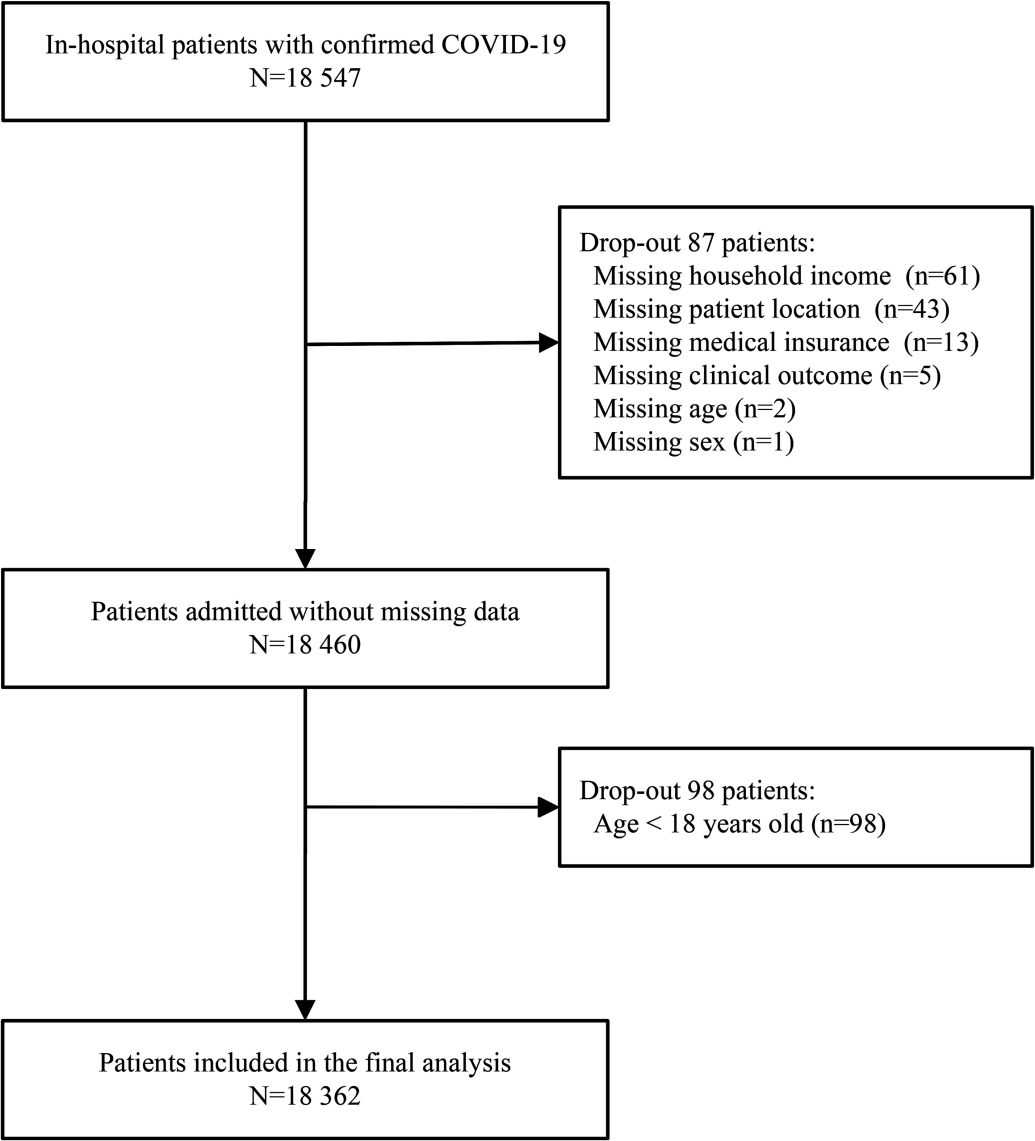
Flow chart of study participants.

Hospitalized patients with COVID-19 were identified according to the International Classification of Diseases, 10th revision (ICD-10) code (U071), and patients aged <18 years were excluded. Missing data were observed for seven variables: household income (61 patients), patient location (43 patients), medical insurance (13 patients), clinical outcome (5 patients), age (2 patients), and sex (1 patients) (Figure 1). These records were excluded because the percentage of missing values was <0.47% (87/18,461).

### 2.2 Outcome variable

The primary outcome was whether the inhospital patient with COVID-19 is admitted to ICU beds, which are important but scarce resource for preventing disease progression.

### 2.3 Health needs

Health needs were measured using the Charlson Comorbidity Index (CCI) score of hospitalized patients with COVID-19. Compared with using the number of comorbidities to measure health needs, the comorbidity index, which considered the different impacts of individual comorbidities on healthcare utilization, can better represent the health needs of hospitalized patients with COVID-19.

The rules for calculating the CCI score in this article are based on the version proposed by Mary E. Charlson, which include 19 comorbidities, with assigned weights ranging from 1 to 6 for each comorbidity (26). A total CCI score was obtained by summing the weights from patient’s medical record of comorbidities, with higher scores indicating more severe comorbid conditions and greater needs.

### 2.4 Covariates

Age, sex, race, household income and medical insurance were selected as covariates for their potential impacts on health service utilization during the pandemic (34, 35). Racial categorization into white and non-white (black, Hispanic, Asian or Pacific Islander, Native American and other) is prevalent due to numerous studies using whites as the reference and have found potential inequities in healthcare service utilization between whites and non-whites. Household income was categorized by quartile classification and identified bya values from 1 to 4, indicating the poorest to wealthiest populations. As previous studies have shown that differences in patient residence and pandemic stage impact the utilization of health services (36, 37), these two variables were also selected as covariates.

### 2.5 Statistical analysis

Continuous variables were presented as mean (standard deviation [SD]) or median (interquartile range [IQR]), and categorical variables as frequencies (%). The demographic and clinical characteristics of the patients were assessed using the Kruskal–Wallis test, Chi-square test, or Fisher’s exact test, as appropriate. To determine whether there is a confounding effect between the independent variables, the following variables were analyzed using Pearson correlation analysis: CCI, sex, age (18–59, 60–69, 70–79, or ≥ 80 years), race (white or non-white), patient location (metropolitan or non-metropolitan areas), medical insurance (Medicare/Medicaid, private insurance, or other), household income (quartiles 1–4), and discharge quarter.

Logistic regression analysis was used to analyze the independent factors that might associated with the equity of ICU admission, and the results were reported as coefficients and odds ratios (OR) with 95% confidence intervals (CI). Variables were assessed by univariable regression first, those with *p* < 0.10 in univariable logistic regression were subsequently entered into multivariable logistic regression (enter method). Additionally, the Shapley value decomposition approach was implemented to assess the contribution of independent variables to disparities in ICU admissions (36). Logistic regression analysis was also initially used to evaluate vertical equity of ICU utilization among hospitalized patients with COVID-19, which was achieved if patients with greater CCI had higher odds of ICU admission.

Then, the vertical equity of health utilization was examined using a concentration curve and concentration index (38). The concentration curve plots the cumulative proportion of ICU admissions (y-axis) against the cumulative proportion of the population ranked according to health needs, beginning with low-need individuals (lower CCI) and ending with high-need patients (higher CCI). The 45-degree line was defined as the line of equity. The concentration curve above or below the line of equity indicated that health utilization was more concentrated among the low-or high-need groups, respectively. The curve reflects whether ICU services are more inclined toward high-demand patients under the condition of bed scarcity among eligible patients. The concentration index was defined as twice the area between the concentration curve and the line of equity and bounded between-1 and 1. It took a positive value when the concentration curve lay below the line of equity, indicating a distribution of health utilization in favor of high needs, and a negative value when the concentration curve lay above the line of equity, indicating a distribution of health utilization in favor of low needs.

Based on the results of these analyses, key factors for further subgroup analysis, including sex, age, race, patient location, medical insurance type, household income, and discharge quarter. For each subgroup, interaction terms between CCI and the categorical variables of interest (e.g., CCI and sex) were introduced into the logistic regression model. Moreover, the concentration index was calculated for each subgroup to do the group-wise comparisons. A *p*-value of less than 0.05 was considered indicative of statistically significant differences in the effect of CCI on ICU utilization and inequality between subgroups, respectively. All statistical analyses were performed using STATA version 16.0 (StataCorp, College Station, TX, USA).

## 3 Results

### 3.1 Demographic and clinical characteristics

A total of 18,547 patients with confirmed COVID-19 were screened for eligibility, and 18,362 patients were included in the final analysis (Figure 1). The median age was 63 years (IQR, 51–75), and 10,534 (57.37%) patients were aged ≥80 years. Among these patients, 9,471 (51.58%) were male, 5,921 (32.25%) were white race, and 16,117 (87.77%) lived in metropolitan areas. There were 5,289 (28.80%) patients covered by Medicare or Medicaid, while 6,150 (33.49%) patients belonged to the poorest households. The median CCI was 1 (IQR, 0–3), and 3,490 (19.01%) patients were treated in the ICU. The most prevalent comorbidities were uncomplicated hypertension (42.45%), obesity (30.27%), and diabetes with chronic complications (26.96%). Other baseline characteristics of the patients were presented in Table 1.

**Table 1:** Demographic and clinical characteristics of patients admitted to hospital with COVID-19.

Characteristics	Overall (*n* = 18,362)	ICU admission		*p*
		No (*n* = 14,872)	Yes (*n* = 3,490)	
Sex				<0.001
Male	9,471 (51.58%)	7,537 (50.68%)	1934 (55.42%)	
Female	8,891 (48.42%)	7,335 (49.32%)	1,556 (44.58%)	
Age (median, IQR)	63 (51,75)	63 (50,75)	64 (52,74)	0.029
18–59y	7,828 (42.63%)	6,418 (43.15%)	1,410 (40.40%)	
60–69y	4,078 (22.21%)	3,264 (21.95%)	814 (23.32%)	
70–79y	3,462 (18.85%)	2,744 (18.45%)	718 (20.57%)	
≥80y	2,994 (16.31%)	2,446 (16.45%)	548 (15.70%)	
Died				<0.001
No	16,776 (91.36%)	14,079 (94.67%)	2,697 (77.28%)	
Yes	1,586 (8.64%)	793 (5.33%)	793 (22.72%)	
LOS (median, IQR)/days	5 (3,8)	4 (2,7)	8 (4,15)	<0.001
Expenditure (median, IQR)/USD	14287.00 (8623.25,24622.75)	12864.00 (8055.00,20658.25)	29231.00 (14083.00,61327.00)	<0.001
Race				<0.001
White	5,921 (32.25%)	4,947 (33.26%)	974 (27.91%)	
Non-white	12,441 (67.75%)	9,925 (66.74%)	2,516 (72.09%)	
Patient location				<0.001
Metropolitan areas	16,117 (87.77%)	12,906 (86.78%)	3,211 (92.01%)	
Non-metropolitan areas	2,245 (12.23%)	1966 (13.22%)	279 (7.99%)	
Medical insurance				<0.001
Medicare/Medicaid	11,303 (61.56%)	9,261 (62.27%)	2042 (58.51%)	
Private insurance	5,289 (28.80%)	4,284 (28.81%)	1,005 (28.80%)	
Other	1770 (9.64%)	1,327 (8.92%)	443 (12.69%)	
Household income				<0.001
Quartile 1	6,150 (33.49%)	5,253 (35.32%)	897 (25.70%)	
Quartile 2	5,597 (30.48%)	4,563 (30.68%)	1,034 (29.63%)	
Quartile 3	3,845 (20.94%)	2,960 (19.90%)	885 (25.36%)	
Quartile 4	2,770 (15.09%)	2096 (14.09%)	674 (19.31%)	
Discharge quarter				<0.001
Second quarter	6,861 (37.37%)	5,438 (36.57%)	1,423 (40.77%)	
Third quarter	3,003 (16.35%)	2,351 (15.81%)	652 (18.68%)	
Fourth quarter	8,498 (46.28%)	7,083 (47.63%)	1,415 (40.54%)	
Number of comorbidities (median, IQR)	3 (2,4)	3 (1,4)	3 (2,5)	<0.001
≤3	11,313 (61.61%)	9,423 (63.36%)	1890 (54.15%)	
>3	7,049 (38.39%)	5,449 (36.64%)	1,600 (45.85%)	
CCI (median, IQR)	1 (0,3)	1 (0,3)	2 (1,3)	<0.001
0	5,504 (29.97%)	4,681 (31.48%)	823 (23.58%)	
1–2	7,414 (40.38%)	5,999 (40.34%)	1,415 (40.54%)	
3–4	3,084 (16.80%)	2,382 (16.02%)	702 (20.11%)	
≥5	2,360 (12.85%)	1810 (12.17%)	550 (15.76%)	
AIDS	237 (1.29%)	195 (1.31%)	42 (1.20%)	0.671
Alcohol abuse	384 (2.09%)	301 (2.02%)	83 (2.38%)	0.211
Deficiency anemias	3,572 (19.45%)	2,692 (18.10%)	880 (25.21%)	<0.001
Arthropathies	528 (2.88%)	428 (2.88%)	100 (2.87%)	1
Chronic blood loss anemia	76 (0.41%)	59 (0.40%)	17 (0.49%)	0.547
Leukemia	105 (0.57%)	82 (0.55%)	23 (0.66%)	0.526
Lymphoma	120 (0.65%)	95 (0.64%)	25 (0.72%)	0.693
Metastatic cancer	181 (0.99%)	147 (0.99%)	34 (0.97%)	1
Solid tumor without metastasis, malignant	300 (1.63%)	237 (1.59%)	63 (1.81%)	0.416
Cerebrovascular disease	749 (4.08%)	588 (3.95%)	161 (4.61%)	0.085
Congestive heart failure	2,439 (13.28%)	1849 (12.43%)	590 (16.91%)	<0.001
Coagulopathy	1745 (9.50%)	1,334 (8.97%)	411 (11.78%)	<0.001
Dementia	1895 (10.32%)	1,540 (10.36%)	355 (10.17%)	0.773
Depression	1959 (10.67%)	1,585 (10.66%)	374 (10.72%)	0.944
Diabetes with chronic complications	4,950 (26.96%)	3,729 (25.07%)	1,221 (34.99%)	<0.001
Diabetes without chronic complications	2,469 (13.45%)	2002 (13.46%)	467 (13.38%)	0.922
Drug abuse	351 (1.91%)	297 (2.00%)	54 (1.55%)	0.093
Hypertension, complicated	4,670 (25.43%)	3,607 (24.25%)	1,063 (30.46%)	<0.001
Hypertension, uncomplicated	7,795 (42.45%)	6,364 (42.79%)	1,431 (41.00%)	0.057
Liver disease, mild	1,053 (5.73%)	826 (5.55%)	227 (6.50%)	0.033
Liver disease, moderate to severe	112 (0.61%)	86 (0.58%)	26 (0.74%)	0.309
Chronic pulmonary disease	4,016 (21.87%)	3,180 (21.38%)	836 (23.95%)	0.001
Neurological disorders affecting movement	384 (2.09%)	314 (2.11%)	70 (2.01%)	0.744
Neurological disorders unaffecting movement	1,370 (7.46%)	984 (6.62%)	386 (11.06%)	<0.001
Seizures and epilepsy	739 (4.02%)	601 (4.04%)	138 (3.95%)	0.851
Obesity	5,559 (30.27%)	4,344 (29.21%)	1,215 (34.81%)	<0.001
Paralysis	683 (3.72%)	522 (3.51%)	161 (4.61%)	0.002
Peripheral vascular disease	816 (4.44%)	624 (4.20%)	192 (5.50%)	0.001
Psychoses	800 (4.36%)	661 (4.44%)	139 (3.98%)	0.247
Pulmonary circulation disease	555 (3.02%)	385 (2.59%)	170 (4.87%)	<0.001
Renal failure, moderate	2,135 (11.63%)	1703 (11.45%)	432 (12.38%)	0.131
Renal failure, severe	1,295 (7.05%)	1,001 (6.73%)	294 (8.42%)	0.001
Hypothyroidism	2,102 (11.45%)	1711 (11.50%)	391 (11.20%)	0.636
Other thyroid disorders	333 (1.81%)	268 (1.80%)	65 (1.86%)	0.865
Peptic ulcer with bleeding	83 (0.45%)	56 (0.38%)	27 (0.77%)	0.003
Valvular disease	811 (4.42%)	639 (4.30%)	172 (4.93%)	0.112
Weight loss	634 (3.45%)	480 (3.23%)	154 (4.41%)	0.001

ICU, intensive care unit; IQR, interquartile range; LOS, length of stay; CCI, Charlson Comorbidity Index.

### 3.2 Factors influencing ICU admission: results of logistic regression analysis

The results of the Pearson correlation analysis (Supplementary Table S1) show that, except for a fair association between age and medical insurance (*r* = −0.404), all independent variables are negligibly associated (−0.3 < *r* < 0.3) (39).

ICU admission was significantly affected by CCI, with ORs 1.09 (95% CI: 1.07–1.10; *p* < 0.001) in univariable analysis and 1.11 (95% CI: 1.09–1.13; *p* < 0.001) in multivariable regression analysis, indicating that patients with higher CCI were more likely to be treated in ICU (Table 2).

**Table 2:** Logistic regression and decomposition of correlates for ICU admission in patients with COVID-19.

	Univariable logistic regression		Multivariable logistic regression		Shapley value decomposition	
	OR (95% CI)	*p*	OR (95% CI)	*p*	Shapley value	Contribution
Sex					0.00125	4.56%
Male	Reference		Reference			
Female	0.83 (0.77–0.89)	<0.001	0.85 (0.78–0.91)	<0.001		
Age					0.00133	4.83%
18–59	Reference		Reference			
60–69	1.14 (1.03–1.25)	0.01	1.17 (1.06–1.29)	0.003		
70–79	1.19 (1.08–1.32)	0.001	1.31 (1.17–1.47)	<0.001		
≥80	1.02 (0.91–1.14)	0.725	1.08 (0.95–1.23)	0.218		
Race					0.00173	6.29%
White	Reference		Reference			
Non-white	1.29 (1.19–1.40)	<0.001	1.25 (1.15–1.37)	<0.001		
Patient location					0.00259	9.42%
Metropolitan areas	Reference		Reference			
Non-metropolitan areas	0.57 (0.50–0.65)	<0.001	0.75 (0.65–0.86)	<0.001		
Medical insurance					0.00286	10.41%
Medicare/Medicaid	Reference		Reference			
Private insurance	1.06 (0.98–1.16)	0.147	1.18 (1.07–1.30)	0.001		
Other	1.51 (1.35–1.7)	<0.001	1.63 (1.43–1.86)	<0.001		
Household income					0.00885	32.27%
Quartile 1	Reference		Reference			
Quartile 2	1.33 (1.20–1.46)	<0.001	1.26 (1.14–1.40)	<0.001		
Quartile 3	1.75 (1.58–1.94)	<0.001	1.71 (1.54–1.91)	<0.001		
Quartile 4	1.88 (1.68–2.11)	<0.001	1.85 (1.64–2.09)	<0.001		
Discharge quarter					0.00256	9.33%
Second quarter	Reference		Reference			
Third quarter	1.06 (0.96–1.18)	0.276	1.07 (0.96–1.19)	0.239		
Fourth quarter	0.76 (0.7–0.83)	<0.001	0.84 (0.77–0.91)	<0.001		
CCI	1.09 (1.07–1.10)	<0.001	1.11 (1.09–1.13)	<0.001	0.00628	22.89%

ICU, intensive care unit; OR, odds ratio; CI, confidence interval; CCI, Charlson Comorbidity Index, Charlson.

Compared to patients aged <60 years, the ORs for those aged 60–69 years, 70–79 years, and ≥ 80 years were 1.17 (95% CI: 1.06–1.29; *p* = 0.003), 1.31 (95% CI: 1.17–1.47; *p* < 0.001), and 1.08 (95% CI: 0.95–1.23; *p* = 0.218), respectively. Non-white patients (black, Hispanic, Asian or Pacific Islander, Native American and other) had higher probability of utilizing ICU beds (OR 1.25, 95% CI: 1.15–1.37; *p* < 0.001) than white patients. Patients living in non-metropolitan areas had lower probability of ICU admission (OR 0.75, 95% CI: 0.65–0.86; *p* < 0.001) than those living in metropolitan areas. Compared to patients with Medicare/Medicaid, those with private insurance had higher odds of ICU admission (OR 1.18, 95% CI: 1.07–1.30; *p* = 0.001). As the household income increased, the odds of receiving treatment in the ICU gradually increased. Compared to the poorest patients (Quartile 1), the wealthiest patients (Quartile 4) had highest odds of ICU admission (OR 1.85, 95% CI: 1.64–2.09; *p* < 0.001). Other factors, including sex and discharge quarter, were also significantly associated with ICU admission.

In the Shapley value decomposition analysis, the contribution of household income was 32.27%, followed by the CCI (22.89%), medical insurance (10.41%), patient location (9.42%), discharge quarter (9.33%), race (6.29%), age (4.83%), and sex (5.56%) (Table 2).

### 3.3 Concentration curve and concentration index

**Figure 2:**
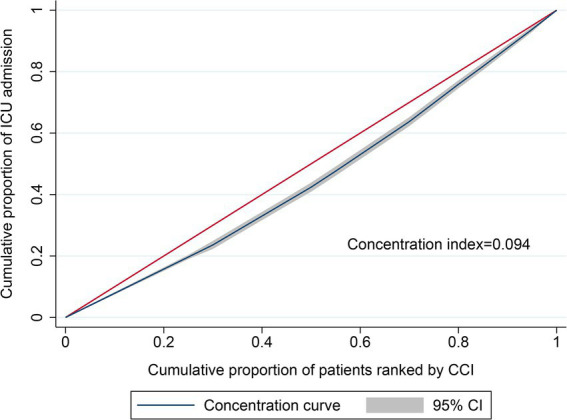
Concentration curve and concentration index of ICU utilization based on CCI.

The concentration curve for ICU admission was below the line of equity and the concentration index was 0.094 (95% CI: 0.076–0.111; *p* < 0.001) (Figure 2), indicating that ICU admission was more concentrated among patients with a higher CCI.

### 3.4 Subgroup analyses

**Figure 3:**
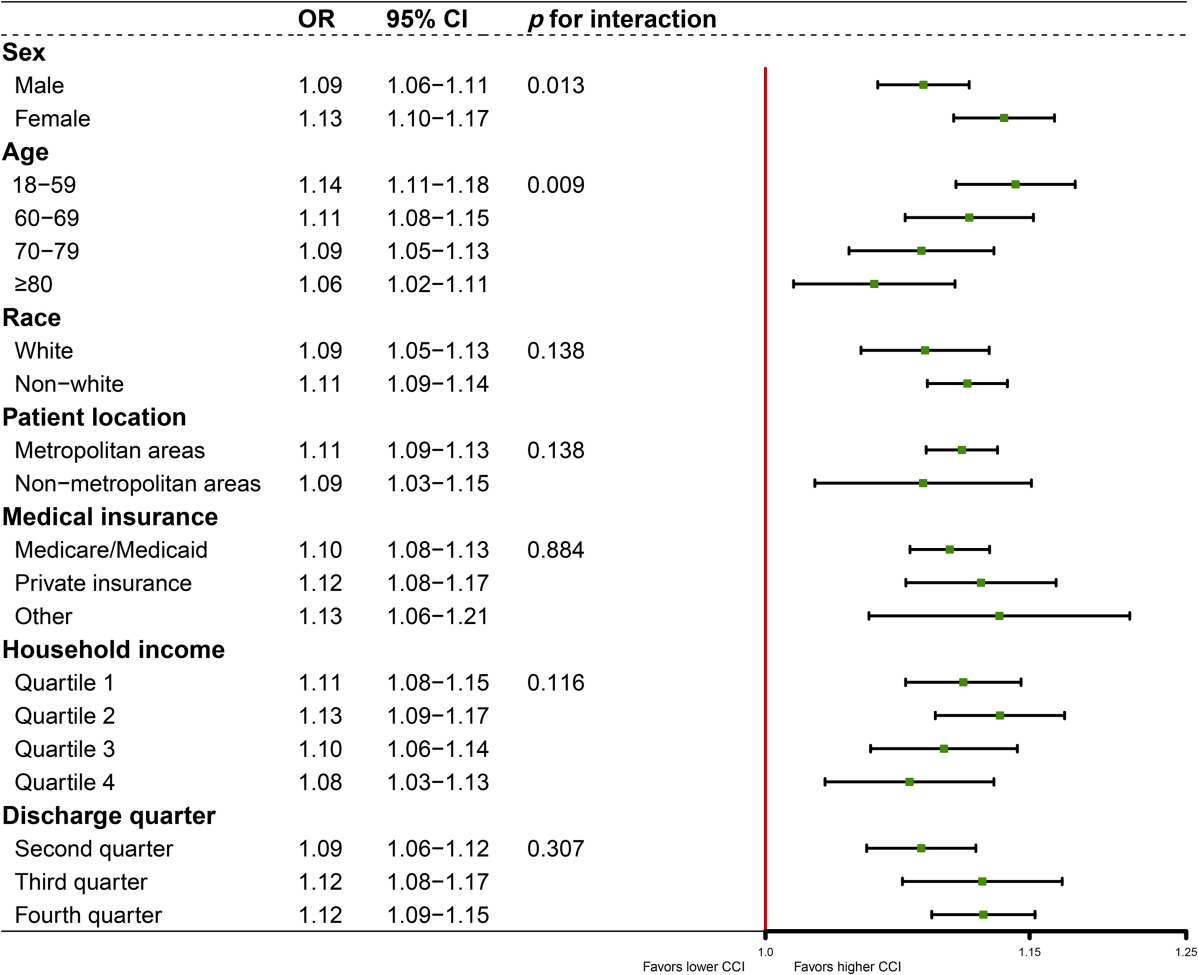
Association between CCI and ICU utilization according to subgroups.

CCI was significantly associated with increased odds of ICU admission in all subgroup analyses by logistic regression, with ORs ranging from 1.06 (95% CI: 1.02–1.11; *p* < 0.001) to 1.14 (95% CI: 1.11–1.18; *p* < 0.001), consistent with results in Table 2. The *p*-value for the interaction suggested sex differences in the association of CCI with ICU admission (*p* = 0.013), with ORs of 1.09 (95% CI 1.06–1.11; *p* < 0.001) for males and 1.13 (95% CI 1.10–1.17; *p* < 0.001) for females (Figure 3). Significant interaction also existed between CCI and age (*p* = 0.009), with ORs 1.14 (95% CI 1.11–1.18; *p* < 0.001) in patients aged <60 years, 1.11 (95% CI 1.08–1.15; *p* < 0.001) in patients aged 60–69 years, 1.09 (95% CI 1.05–1.13; *p* < 0.001) in patients aged 70–79 years, and 1.06 (95% CI 1.02–1.11; *p* < 0.001) in patients aged ≥80 years. No significant interactions were observed in the subgroup analyses by race (*p* = 0.138), patient location (*p* = 0.138), medical insurance (*p* = 0.884), household income (*p* = 0.116), or discharge quarter (*p* = 0.307).

**Figure 4:**
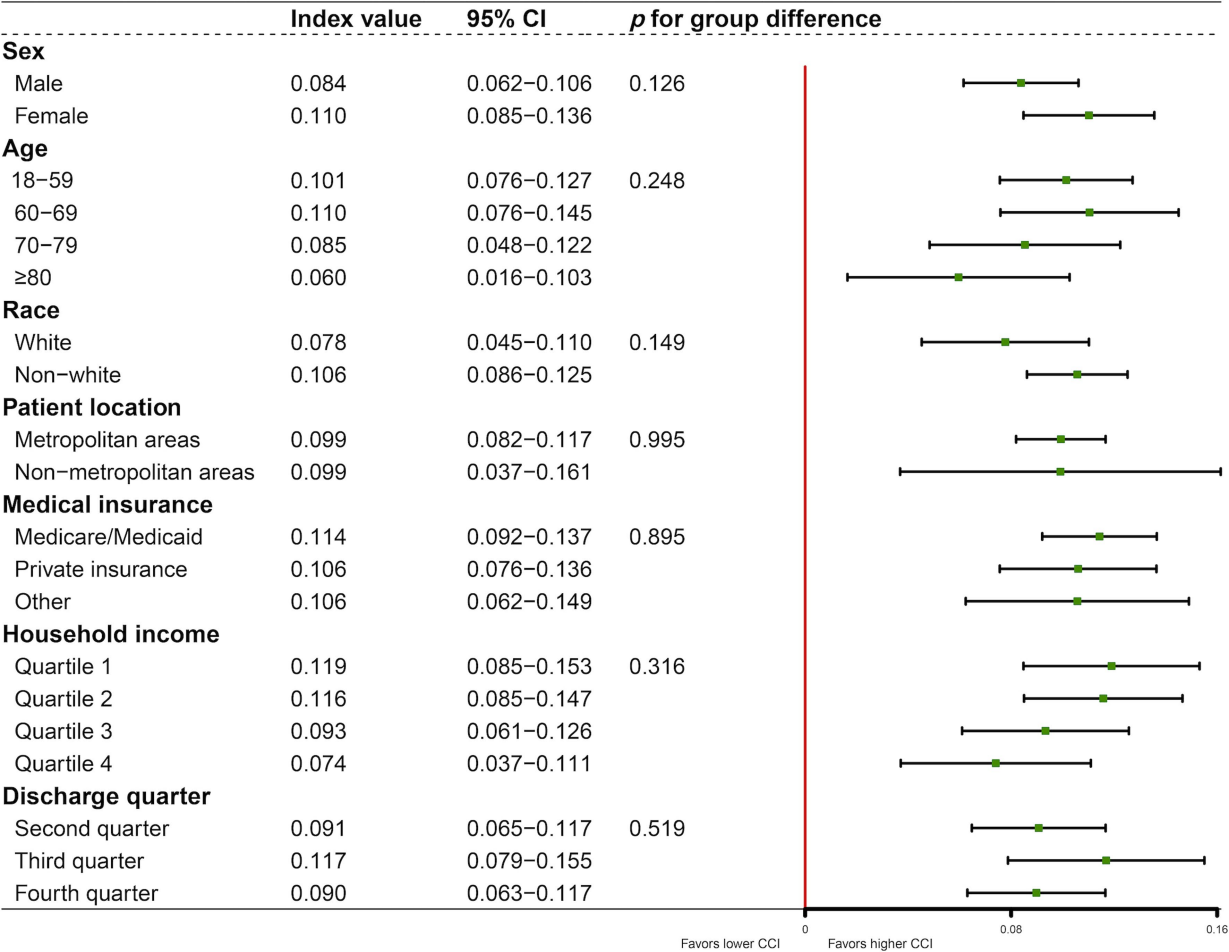
Subgroup analyses of concentration index.

In the subgroup analyses, the concentration index of ICU utilization ranged from 0.060 (95% CI: 0.016–0.103; *p* = 0.007) to 0.119 (95% CI: 0.085–0.153; *p* < 0.001) (Figure 4), implying the distribution of ICU admission in favor of patients with higher CCI. There were no significant differences between the subgroups in terms of sex (*p* = 0.126), age (*p* = 0.248), race (*p* = 0.149), patient location (*p* = 0.995), medical insurance (*p* = 0.895), household income (*p* = 0.316), or discharge quarter (*p* = 0.519).

## 4 Discussion

Vertical equity is defined as people with higher needs receiving more healthcare (40, 41). In this study, we attempted to evaluate the vertical equity of ICU admissions for COVID-19 patients in the early stage of the pandemic by using CCI to represent patients’ health needs. Through logistic regression analysis and a concentration index/concentration curve, this study comprehensively analyzed the vertical equity of ICU utilization among 18,362 hospitalized patients with COVID-19 in Maryland. The results showed that ICU utilization was more concentrated in patients with higher CCI, which was consistent with the principle of vertical equity. Subgroup analysis were also conducted to verify the robustness of the results.

In this study, it was found that in addition to CCI, other factors including household income, health insurance, patient location, race, age, and sex were all associated with ICU utilization. Among them, Shapley value decomposition analysis suggested that household income (32.27%) contributed even more to ICU utilization than CCI (22.89%). This result is consistent with the findings of Khanijahani et al. (42), indicating that socioeconomic status has a significant effect on healthcare utilization. COVID-19 hospitalized patients with higher socioeconomic status (having private insurance or higher household income) were more likely to use the ICU.

Age was one of the most important factors influencing healthcare utilization. Due to older patients may have more comorbidities and poor basic health status (43), some studies used age as an alternative assessment of health needs, suggesting that older patients required more healthcare services (42, 44). In this study, compared to patients aged 18–59 years, the probability of ICU utilization among patients aged ≥80 years did not significantly increase. However, interaction analysis also suggests that increasing age significantly weakened the effect of CCI on ICU utilization. In the subgroup analysis of logistic regression, the OR of CCI on ICU utilization decreased from 1.14 (18–59 years) to 1.11 (60–69 years), 1.09 (70–79 years) and 1.06 (≥ 80 years), and similar decreases were also found in the subgroup analysis of concentration index. This might be explained by the following reasons. First, patients’ willingness to choose aggressive treatment (especially invasive treatments that often occur in the ICU) decreased as age increased (45, 46). Second, when considering the potential complications and patient survival probability of implementing aggressive treatment, clinicians were unlikely to recommend such treatment to older patients (47). Third, the allocation of scarce medical resources (ICU, ventilators, etc.) was more likely to favor young patients with a higher probability of survival (48).

In our study, sex was another important factor influencing ICU admission and the correlation between CCI and ICU. In the initial logistic regression, the OR of 0.83 for women compared to men reflects a direct comparison between the two sexes, with men as the reference group. This suggests that among COVID-19 patients, females are less likely to be admitted to the ICU than males when no other variables are considered, which is consistent with previous findings (49, 50). However, in the subgroup analysis, females instead strengthened the association between CCI and ICU (1.13 for female and 1.09 for male). This may be due to the fact that females were more sensitive to CCI when it was used as a measure of health need, with each one-unit increase in CCI increasing the odds of ICU admission for females more than for males. Thus, although females are more sensitive to changes in units, the lower rate of utilization among females than males does not conflict with the status quo.

However, this study has some limitations. First, disease severity is a critical factor influencing ICU utilization. Due to limitations in data availability, we were unable to include it as a control variable in our analysis. This omission may impact the robustness of our findings and suggests caution in interpreting the results. Second, as this study is based on cross-sectional data collected at a single time point, establishing causal relationships between the variables analyzed is inherently challenging. The temporal limitations of the data restrict our ability to make definitive causal inferences. Third, this study was conducted using data from hospitalized COVID-19 patients in Maryland only, which limits the generalizability of our findings. The results may not be applicable to other regions or populations, particularly those with different healthcare infrastructures, patient demographics, or COVID-19 management strategies.

## 5 Conclusion

The results of this study demonstrated that ICU utilization was more concentrated among patients with higher health needs in the early stages of the pandemic, indicating vertical equity in healthcare utilization during this period. For high-risk groups such as those with a high burden of comorbidities, policy makers should consider their urgent need for health services when implementing public health intervention measures, and reduce the inequity of healthcare utilization during the pandemic.

## Data Availability

The original contributions presented in the study are included in the article/Supplementary material, further inquiries can be directed to the corresponding author.
