# Supplementary material for: Equity evaluation of intensive care unit admission based on comorbidity in hospitalized patients with COVID-19: a cross-sectional analysis
Source: Front Public Health. 2024 Oct 28;12:1430462. doi: 10.3389/fpubh.2024.1430462 (PMC11550993; doi:10.3389/fpubh.2024.1430462)
Supplement: Supplementary file 1 [file Table_1.docx]

**Table 1** **Correlation analysis between variables between CCI and control variables**

| Variables | Sex | Age | Race | Patient  location | Medical  insurance | Household  insurance | Discharge  quarter | CCI |
| --- | --- | --- | --- | --- | --- | --- | --- | --- |
| Sex | 1.000 |  |  |  |  |  |  |  |
| Age | 0.077 | 1.000 |  |  |  |  |  |  |
| Race | 0.003 | -0.295 | 1.000 |  |  |  |  |  |
| Patient location | -0.009 | 0.078 | -0.253 | 1.000 |  |  |  |  |
| Medical insurance | -0.078 | -0.404 | 0.155 | -0.074 | 1.000 |  |  |  |
| Household income | -0.026 | 0.083 | -0.088 | -0.181 | 0.042 | 1.000 |  |  |
| Discharge quarter | 0.008 | 0.096 | -0.204 | 0.126 | -0.082 | -0.002 | 1.000 |  |
| CCI | 0.013 | 0.308 | -0.047 | 0.056 | -0.252 | -0.041 | 0.006 | 1.000 |
|  | | | | | | | | |
